# An *Arabidopsis* mutant line lacking the mitochondrial calcium transport regulator MICU shows an altered metabolite profile

**DOI:** 10.1080/15592324.2023.2271799

**Published:** 2023-10-25

**Authors:** Emilia R. Gutiérrez-Mireles, José Carlos Páez-Franco, Raúl Rodríguez-Ruíz, Juan Manuel Germán-Acacio, M. Casandra López-Aquino, Manuel Gutiérrez-Aguilar

**Affiliations:** a Departamento de Bioquímica, Facultad de Química, Universidad Nacional Autónoma de México, Ciudad de México, México; b Red de Apoyo a la Investigación, Coordinación de la Investigación Científica-UNAM, Instituto Nacional de Ciencias Médicas y Nutrición Salvador Zubirán, Ciudad de México, México

**Keywords:** Mitochondrial calcium uniporter, *Arabidopsis thaliana*, plant metabolism

## Abstract

Plant metabolism is constantly changing and requires input signals for efficient regulation. The mitochondrial calcium uniporter (MCU) couples organellar and cytoplasmic calcium oscillations leading to oxidative metabolism regulation in a vast array of species. In *Arabidopsis thaliana*, genetic deletion of AtMICU leads to altered mitochondrial calcium handling and ultrastructure. Here we aimed to further assess the consequences upon genetic deletion of AtMICU. Our results confirm that AtMICU safeguards intracellular calcium transport associated with carbohydrate, amino acid, and phytol metabolism modifications. The implications of such alterations are discussed.

## Introduction

In *Arabidopsis thaliana*, calcium ions regulate signaling and metabolism under normal or stress conditions.^1^ While it is well established that this divalent cation can alter the fate of a plant cell, the mechanisms by which plant cells transport Ca^2+^ are still under study.^2^ Intracellular calcium buffering and storage play important roles in the homeostasis of this cation. Consequently, organellar calcium transport and sensing lays at the center of plant metabolism following multivariate physiological and pathological cues.^3,4^ Intracellular calcium signaling sensors are important for shoot and root development as well as for pollen tube, stomatal and hormonal homeostasis.^5,6^ These signaling proteins orchestrate plant physiological responses through protein post translational modifications leading to changes in metabolism and cell structure.^7–9^ Several transporters allowing calcium to enter a given organelle including Golgi apparatus.^10^, chloroplasts,^11,12^ ER^13^ and mitochondria^14^ have been unveiled during the last decades. The activity of some of these carriers can be detected upon specific signals. For example, darkness can induce calcium transients in *Arabidopsis* seedling thylakoids and stromata in a process coupled to circadian rhythms.^15,16^ Mitochondrial calcium levels can oscillate around 200 nM under resting conditions^17–19^ and markedly upsurge in response to cold, osmolarity, mechanic stimuli (i.e., touch, presence of herbivores, etc.) and reactive oxygen species (ROS).^20–24^ Extracellular calcium oscillations have been carefully studied and proposed to constitute an effective signal -alongside ROS bursts- allowing plants to prepare for an upcoming challenge sensed by a small number of cells.^25^ Plant mitochondrial calcium ions are imported through different transporters including a putative glutamate induced Ca^2+^-permeable AtGLR3.5^14^ and a ruthenium red (RuR)- sensitive MCUc.^11^ Mitochondrial Calcium Uniporter (MCU) holocomplexes are formed by calcium-transporting tetramers of MCU subunits spanning inner mitochondrial membranes coupled to a calcium-binding ‘gate-like’ MICU subunit in the intermembrane space.^26^ While this holocomplex is known to transport calcium ions, some studies have reported the ability to transport Mn^2+^ cations.^27,28^ In *A. thaliana*, six MCU isoforms (AtMCU) and only one MICU homolog (AtMICU) constitute the minimal known configuration of MCUc.^29–31^ Plant lines lacking one of the channel-forming MCU isoforms were found to feature distorted mitochondrial ultrastructure and altered primary root length^31^ (absence of AtMCU1) and impaired pollen tube germination and growth^29^ (absence of AtMCU2). Plants lacking channel-forming AtMCU1 isoform display distorted mitochondrial ultrastructure, altered primary root size^31^ and impaired pollen tube germination and growth^29^ alongside changes in calcium levels. Plants lacking chloroplast-specific MCU can display drought tolerance.^12^ In addition, triple mutant plants lacking AtMCU1, 2 and 3 present a drastic reduction in mitochondrial calcium transport and show deregulated jasmonic acid-related signaling.^32^ Conversely, plants lacking AtMICU show alterations in mitochondrial calcium levels and organellar ultrastructure, while overall plant morphology appears unchanged.^30^ Here we aimed to further assess the physiological consequences of plants lacking AtMICU alongside potential derangements on overall metabolism derived from altered intracellular calcium transport. Our results confirm that AtMICU has an inhibitory effect on RuR-sensitive Ca^2+^ uptake into the mitochondria. They further indicate that lack of AtMICU has consequences that affect amino acid and carbohydrate metabolism.

## Materials and methods

### Materials

Digitonin, succinate, mannitol, MES, anhydrous CaCl_2_, H_3_BO_3_, sucrose, KH_2_PO_4_, RuR and Evans blue were purchased from Sigma-Aldrich. Calcium Green-5N was purchased from Invitrogen. All chemicals used for metabolomic analysis were HPLC grade.

### Plant materials

Isogenic *Arabidopsis thaliana* Col-0 and T3 sequence-confirmed *micu-1* (SALK_064052C) lines were obtained from the Arabidopsis Biological Resource Center, Ohio State University, USA. In this study, we decided to use this single *micu-1* mutant strain since it belongs to the SALK Confirmed T-DNA Project^33^ where T-DNA insertion was sequence-indexed at the AT4G32060 locus. TDNA-Seq is a pipeline for next-generation sequencing where pools of T-DNA lines can be sequenced in parallel and then de-convoluted based on their pooling pattern to assign T-DNA insertions back to their originating seed line. AT4G32060 gene interruption was validated through standard genotyping (Fig. S1). Seeds were routinely sterilized with 50% (v/v) bleach, 0.02% Tween 20 for 10 minutes before placing in vertical plates containing 1/2× MS media plus 1% agar supplemented with 0.1% (w/v) sucrose. Plates were stratified at 4°C under dark conditions for 72 h and placed in an Ambi-Hi-Lo incubator at 22°C under a 16 h/8 h light/dark photoperiod. Two-week-old seedlings were transferred to standard pots containing Sunshine Mix #3 (Sun Gro Horticulture), agrolite and perlite (2:1:1) during two additional weeks under standard greenhouse conditions for downstream experiments. One limitation of the present study pertains to the utilization of a single *micu-1* mutant strain to understand potential metabolic adaptations. While this strain presents a similar phenotype to one previously reported (i.e. enhanced calcium uptake^30^ and belongs to the SALK Confirmed T-DNA Project,^33^ it is always a better practice to obtain results and draw conclusions based on same locus mutations in as many available ecotypes.

### Leaf mesophyll protoplast isolation

Leaves (25–30) were treated with 10 mL protoplast enzyme solution (PES) containing 1% cellulase (Sigma Cat., C0615), 0.25% Pectinase (Sigma Cat., P2401), 0.4 M mannitol, 20 mM KCl, 20 mM MES, pH 5.7 under the conditions described by Yoo and colleagues with minor modifications.^34^ Briefly, leaves were vacuum infiltrated in PES for 30 min under dark conditions. Samples were incubated for 90 min at 40rpm and 25°C under dark conditions. Released protoplasts were treated with 10 mL W5 solution containing 154 mM NaCl, 125 mM. CaCl_2_, 5 mM KCl and 2 mM MES, pH 5.7. Protoplasts were used for experiments within a 16 h time frame after each isolation procedure.

### Protoplast permeabilization

Isolated protoplasts were washed twice in Protoplast Assay Buffer (PAB) containing 330mOsM mannitol, 1 mM KH_2_PO_4_, 0.1 mM EDTA, 10 mM MES pH 5.7 and resuspended in PAB containing 0.01% digitonin or not (control) and stirred at 60rpm for 5 minutes. Protoplast permeability was determined under the microscope after staining for 5 minutes with 1% Evans blue followed by extensive washing with PAB.^35^

### Mitochondrial polarization assessment in col-0 and micu-1 leaf mesophylls

Three-week old plants from Col-0 and *micu-1* genotypes were allowed to grow under standard greenhouse conditions at 22°C. Leaf mesophylls from both plants were stained with JC-1 using a previously described method and mitochondrial polarization was assessed under the microscope.^36^

### Calcium transport assessment

Washed permeabilized protoplasts were resuspended in PAB buffer containing 10 mM succinate to a cell density of 2 × 10^5^/mL. After incubating the samples for 5 minutes in a fluorimeter cuvette, 10 μL from a stock solution of 10 mM CaCl_2_ were added to measure calcium uptake in a final volume of 1 mL. Calcium transients were assessed by measuring changes in the fluorescence of 2 μM Calcium Green-5N at 500 nm_ex_/530 nm_em_ as previously reported.^37^ Calcium transport was also assessed in the presence of 2 μM RuR to determine the potential contribution of MCUc to such transport activity.

### Protoplast mitochondrial integrity

Control or permeabilized protoplasts were resuspended in PAB buffer containing 10 mM glutamate to a cell density of 2 × 10^5^ in a final volume of 1 mL and in the presence of 1 μM rhodamine 123. After incubating the samples for 25 minutes at room temperature, mitochondrial morphology and polarity were assessed at 40X magnification using an Olympus B×51epifluorescence microscope with a 460–490 nm_ex_ light source. Mitochondria were detected as bright-green objects with punctate appearance, while chloroplast autofluorescence was monitored in red.

### Untargeted metabolomics analysis

Metabolites were extracted from ~0.16 g fresh leaves of 3-week-old plants from both genotypes with HPLC-grade solvents at −20°C. Plant tissue was weighted in an analytical balance and immediately grinded in a cold mortar with 2 mL MeOH/H_2_O (3:1) at −20°C. Samples were cleared at 12,000 rpm for 10 minutes. Supernatants were transferred to new tubes for downstream analyses. Then, 20 µL of internal standards (tridecanoic acid and 5α-cholestane, 0.18 mg/mL each) were added to the final extract and vortexed for 2 min. A blank consisting of methanol was included to check the presence of contaminants throughout the entire analysis.

The resultant extracts were divided in two equal aliquots and processed independently to identify any possible changes in retention time and intensity values during the protocol.

All extracts were dried until desiccation in a speedvac (Savant SPD121P-Thermo Scientific). For the derivatization process, 80 µL of methoxiamine hydrochloride (Sigma) in pyridine (Sigma) were added to the tube, vortexed and incubated for 90 min at 37°C under agitation. For trimetilsylilation, 80 µL of MBSTFA (Sigma) with 1% TMCS (Sigma) were added an incubated for 30 min at 37°C. Finally, one microliter was injected (splitless) with an autosampler (G4513A – Agilent) in a GC/MS system (5977A/7890B – Agilent) with an HP-5 ms (30 m × 250 µm × 0.25 µm – Agilent) column with helium 99.9999% as a mobile phase and at 1 ml/min flow with the following parameters: 200°C inlet temperature, 200°C source temperature, and 250°C interface temperature. The running method was set to 1 min hold at 60°C with a ramp of 10°C/min until 325°C. All samples were injected stochastically. A quality control sample consisting of a pool mixture of all the samples was injected every 4 samples to identify potential changes in retention times and peak intensities. Only those metabolites with less than 30% of relative standard deviation in the quality control samples were included in the final analysis. The relative standard deviation values for the tridecanoic acid and 5α-cholestane in all the samples analyzed was 8.3% and 7.6% respectively. Alignment and deconvolution were performed in MZmine 2.53 with the following parameters: RT range, 5.5–27.5 min; m/z range, 50–500; m/z tolerance, 0.5; noise level, 1 × 10^3;^ and peak duration range, 0.01–0.2 min.^38^ The ‘rule of 80%’ was implemented for all the samples. For NIST library identification, a match and reverse match > 700 and % probability > 70% were marked as correct. Those metabolites with a probability < 70% but a match and a reverse match > 700 were marked as unknown but included in the final analysis. Most probable IDs were included and metabolomic experiments were done by triplicate and independently.

### Statistical analyses

For PCA, heatmap, hierarchical clustering, and fold change analysis, the data was sum normalized. The internal standard normalization (employing tridecanoic acid) shown similar results. Only those metabolites with fold change > 2 and *p* < 0.05 controlled by FDR were considered significant.

For multivariate analysis (PLS-DA), the data was normalized by sum, mean centered and log transformed in Metaboanalyst 4.0. Metabolic pathway analysis and enrichment pathway analysis were performed in Metaboanalyst 4.0 employing the KEGG Arabidopsis library.

For all other results, data are presented as mean + S.E.M of at least 3 independent biological replicates. Statistical evaluation between 2 groups was performed by unpaired t-tests and a *P* value < 0.05 was considered as criteria of significance.

## Results

### Intracellular calcium transport and mitochondrial polarization in digitonin-permeabilized protoplasts and leaf mesophylls

**Figure 1.**
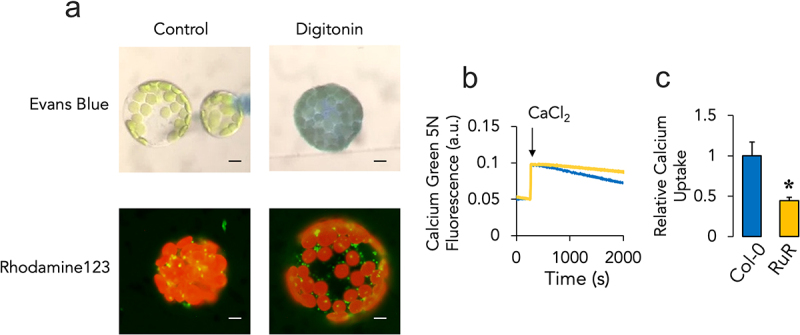
Digitonin-induced membrane permeabilization and intracellular calcium transport in isolated protoplasts from *A. thaliana.* (a) protoplasts (2 × 10.^5^ were isolated as detailed in the methods section and incubated in PAB buffer with 10 mM succinate plus 1% Evans blue or 1 μM rhodamine 123 under control conditions or in the presence of 0.01% digitonin for 5 minutes. Cytoplasmic staining or mitochondrial membrane potential were assessed under the microscope. (b) permeabilized protoplasts (2 × 10.^5^ were incubated with 2 μM calcium Green-5N and challenged with a single 100 μM CaCl_2_ pulse (arrow). Calcium uptake dynamics were then assessed for 2000s in the absence (blue trace) or presence (yellow trace) of 2 μM RuR. (C) relative calcium uptake rates were normalized under control conditions (Col-0) and compared to those in the presence of RuR. Data are presented as mean ± SEM. *p ≤ 0.05 for control versus RuR with *n* ≥ 4 with separate protoplast preparations in different experiments. Bar = 5 μm.

Protoplasts isolated from *Arabidopsis thaliana* leaf mesophylls represent a useful model to study intracellular processes.^34^ To understand the contribution of MCU-dependent intracellular calcium transport in an *in-situ* setting, we first decided to test whether digitonin addition could efficiently permeabilize protoplast plasma membranes (Figure 1a). Under such conditions, addition of membrane-impermeable Evans Blue resulted in an efficiently stained cytoplasm, while chloroplast morphology remained unaffected. Intact and permeabilized isolated leaf mesophyll protoplasts were able to establish a mitochondrial membrane potential (ΔΨ) as assessed by rhodamine 123 staining in the presence of respiratory substrates (Figure 1a). We next decided to test the contribution of MCU on the overall intraorganellar calcium transport activity by monitoring changes in cytoplasmic calcium-dependent Calcium Green 5N fluorescence in the absence or presence of the MCU inhibitor RuR (Figure 1b). As previously reported,^39^ *in situ* transport was assessed after the addition of an initial calcium bolus (evidenced as a fluorescence peak) and monitored through a decrease in fluorescence. A steady, yet slow calcium uptake phenotype was detected in permeabilized protoplasts in the absence of RuR (Figure 1b, blue trace). In the presence of 2 μM RuR however, calcium transport was significantly -but not completely- decreased (Figure 1b, C, yellow trace/bar).

**Figure 2.**
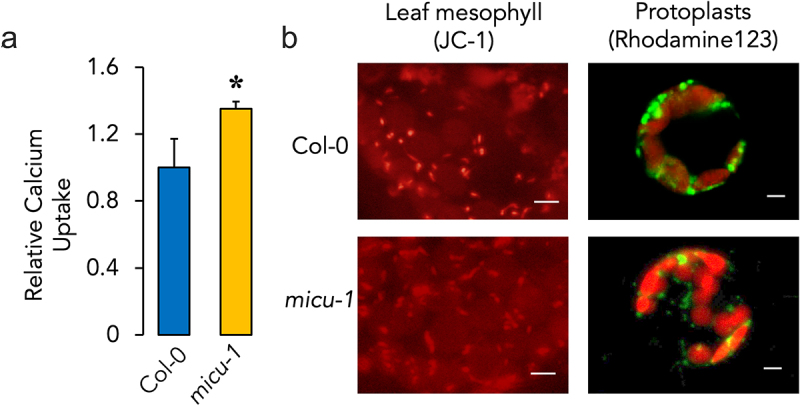
Effects of AtMICU gene knockout on calcium transport and mitochondrial ΔΨ. (a) protoplasts from Col-0 and *micu-1* leaf mesophylls were isolated as detailed in the *methods* section and relative calcium uptake rates were measured for Col-0 protoplasts and compared to those obtained with protoplasts isolated from *micu-1* leaf mesophylls. Data are presented as mean ± SEM. *p ≤ 0.05 for Col-0 versus *micu-1* with *n* ≥ 4 with separate protoplast preparations in different experiments. (b) leaves were stained as detailed in the *mitochondrial polarization assessment in Col-0 and micu-1 plants* section. Isolated protoplasts were stained with rhodamine 123 as in fig. 1. Representative images *N* = 3. Bar = 5 μm.

We next sought to assess potential changes in the calcium transport activity of permeabilized protoplasts isolated from micu-1 leaf mesophylls (Figure 2). Protoplasts from this mutant plant showed significantly enhanced calcium transport rates (Figure 2a). These changes were not related to alterations in mitochondrial ΔΨ since we monitored no apparent changes in leaf mesophyll mitochondrial polarization under baseline conditions on both Col-0 and *micu-1* plants before or after isolating protoplasts (Figure 2b).

### Metabolomics assessment in col-0 and micu-1 leaf mesophyll extracts

**Figure 3.**
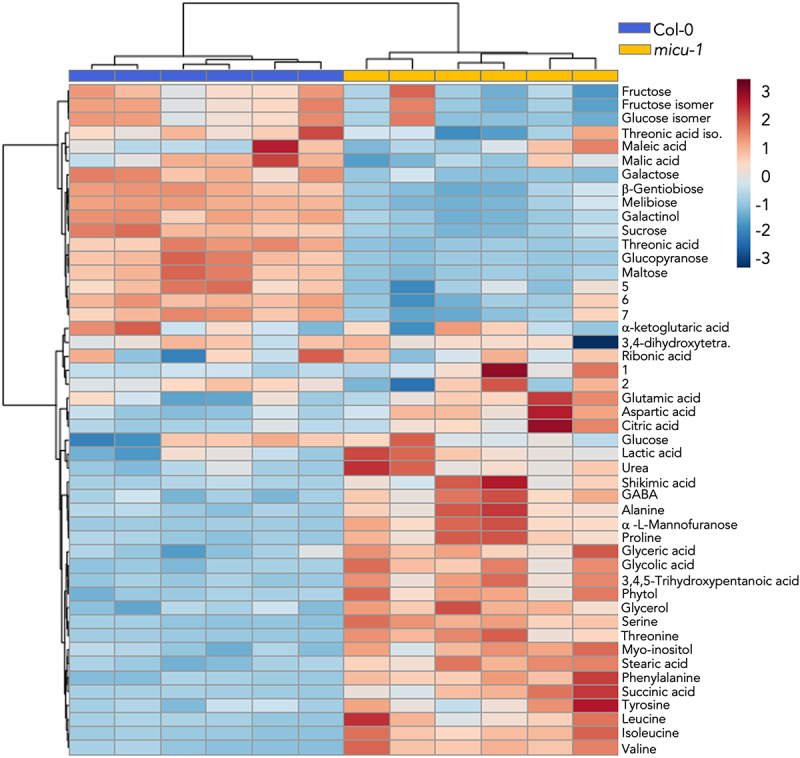
Metabolomics assessment of Col-0 versus *micu-1* leaves. heatmap representation and clustering analysis of differential metabolites detected between Col-0 (blue bar heading) and *micu-1* (yellow bar heading) extracts. Only metabolites with *p <0.05* values as assessed by ANOVA are shown. For Col-0 and *micu-1* metabolite sample groups *N* = 3 and *n* = 6.

**Figure 4.**
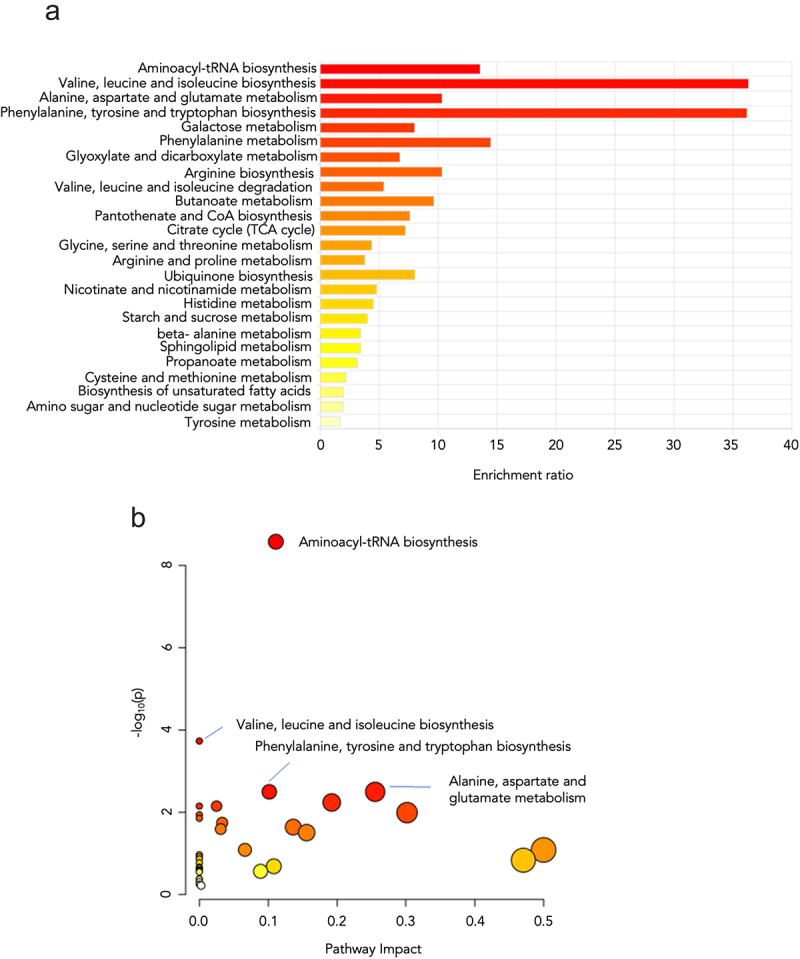
Significant pathway mapping based on the *Arabidopsis thaliana* metabolite library (KEGG). (a) Enriched metabolite sets and b) pathway analysis of differential metabolomic profiles between Col-0 and *micu-1* leaf mesophyll extracts. Only those metabolic pathways with *p* < 0.05 are depicted.

Considering the results presented above showing altered calcium handling in leaf protoplasts from *micu-1* plants, plus those previously published highlighting the occurrence of mitochondrial ultrastructural alterations in the absence of AtMICU,^30^ we sought to determine potential compensatory changes in the metabolism of leaves and roots allowing plants to develop with relative normalcy. While we did not monitor substantial changes on the overall metabolic profile in root extracts from *micu-1* plants (results not shown), we detected variations in several leaf metabolites of micu-1 plants when compared to its isogenic Col-0 counterpart (Figure 3). The results effectively dichotomize the levels of several metabolites between the Col-0 and *micu-1* groups in terms of hierarchical clustering and partial least squares discriminant analysis (PLSDA) (Fig. S2A and B). The variable importance in the projection values show the amino acids and carbohydrates that significantly discriminate in samples from both plant genotypes (Fig. S2B). Our metabolomic approach is robust to quantify the metabolites included in the final analysis as determined by the compact cluster of quality control (QC) samples in the principal component analysis (PCA) assessment (Fig. S3). All significant changes (FC > 2 and *p* < 0.05) in metabolite levels are summarized in Supplementary Table S1. Metabolic pathway analysis and enrichment pathway analysis of metabolites with significant changes suggest that valine, leucine, isoleucine, phenylalanine, tyrosine, tryptophan, alanine, aspartate, glutamate and aminoacyl-tRNA biosynthesis metabolism is altered in samples from *micu-1* plants (Figure 4a,b). In line with this, aromatic amino acid precursor shikimic acid^40^ was found at increased concentrations in *micu-1* plants (Figure 3a).

Furthermore, significant decreases on several carbohydrates in the micu-1 context, involving threose derivative threonic acid were also evident. Desiccation-related galactinol.^41^ was also found at lower levels and chlorophyll related phytol was significantly upregulated (Figure 3a).^42–44^

Krebs cycle intermediates including succinic and citric acids were found at significantly higher levels in extracts from *micu-1* leaves (Table S1). Finally, other metabolites known for its effects on plant growth, stress tolerance and catabolism including GABA,^45^ proline^46^ and urea^47^ were significantly accumulated in *micu-1* plants (Figure 3a, Table S1).

## Discussion

The interrelations of several metabolic pathways with MCUc-mediated calcium transport are well documented in mammalian mitochondria.^48,49^ In line with this, Nemani and collaborators found that fluctuations in mitochondrial metabolism can alter MCUc composition and activity.^50^ Mammalian cells may achieve this either by regulating the expression levels of the MCUc ‘gate-like’ subunit MICU1 in a Krebs cycle- and EGR1-dependent manner^50^ or through Akt-dependent MICU1 phosphorylation.^49,51^ Hence, deletion or silencing of MICU1 exacerbates mitochondrial matrix ɑ-ketoglutarate utilization and NAD(P)H production in HEK293 cells^52^ yet decreases ATP production in diverse contexts.^52,53^ Mammalian MICU1 is also considered a ‘plug’ regulating calcium fluxes upon methylation by PRMT1 during aging or cancer.^54^

Less is known about the molecular cues controlling organellar calcium levels relevant to the overall homeostasis of this cation. However, several transporters mediating calcium import into mitochondria and chloroplasts have been identified.^1,55^ For example, plants lacking MCU1 or MCU2 have been shown to be involved in physiological processes including root and pollen tube growth, as well as stomata-mediated drought stress tolerance.^12,29,31^ Some of these changes are derived from the presence of an MCUc located in the chloroplast inner envelope membranes.^12^ Whether the changes reported herein are derived from the effects of AtMICU on every or specific MCUc isoforms remains to be tested. Moreover, AtMICU has been shown to be relevant for efficient control of calcium fluxes into the mitochondrial matrix and for the maintenance of the ultrastructural features of these organelles.^30^ In agreement with previously reported results,^31^ we found that the absence of AtMICU results in increased intraorganellar calcium uptake. We also monitored metabolic differences between both genotypes, being most evident at the carbohydrate and amino acid levels. Decreased carbohydrate concentrations could well mean metabolic fluxes are shifted toward respiratory substrate production in micu-1 plants. Indeed, TCA cycle succinate and citrate were upregulated in the micu-1 context further indicating carbohydrate carbon scaffolds may accumulate either as mitochondrial intermediates or other carbon skeletons. Lipids such as stearic acid were also significantly upregulated in the micu-1 context. This is of special relevance considering previous studies by Fatland and colleagues linking cytoplasmic ATP-dependent citrate lyase with isoprenoid and fatty acid biosynthesis.^56^ These results are also consistent with the increased concentrations of phytol and stearic acid reported herein. Several amino acid levels were also significantly upregulated, indicating transamination reactions may also be of utmost importance when MCUc-dependent calcium flux is exacerbated (i.e. in the absence of regulatory AtMICU). Considering shikimate levels are upregulated in *micu-1* plants, it is also tempting to speculate that AtMICU-dependent calcium overload can activate shikimate pathway from PEP, thus yielding increased levels of aromatic Phe and Tyr.^57^ The fate of such metabolites could well suggest increased salicylate signaling.^58,59^ Among other metabolites with increased levels, GABA is of special relevance since its accumulation has been associated with the overexpression of anion transporters resulting in changes in pollen tube and root growth,^60^ which would fit with previous findings.^29^

One interesting -yet unexpected- result was the detection of increased phytol levels in *micu-1* plants. This terpene is a known chlorophyll metabolism intermediate and may indicate either leaf senescence or enhanced tocopherol synthesis.^43,44^ We did not monitor any senescent phenotype in *micu-1* plants, and chronic mitochondrial calcium elevation is a known leaf senescence suppressor.^61^ In addition, increased calcium levels can induce de-etiolation and chlorophyll accumulation in *A. thaliana*.^62^ Thus, potential remobilization of phytol from chlorophyll breakdown could well end up in exacerbated tocopherol biosynthesis and enhanced resistance to photooxidative stress.^43,63^ This hypothesis fits well with the enhanced shikimic acid levels found in *micu-1* plants. In addition, enhanced phytol and shikimic acid pathway metabolites (Tyr and Phe) are required for tocopherol production in a phytol kinase-dependent manner.^44,64^ Whether any of these possibilities occur in the *micu-1* context remains to be tested.

In this study, we also detected significantly higher serine levels in *micu-1* plants. Serine is biosynthesized through HPR1-dependent photorespiration (glycolate pathway), the glycerate pathway and the ‘phosphorylated pathway’ in C3 plants like *Arabidopsis*.^65,66^ The latter contributes to a substantial extent to serine production and when genetically suppressed, a strong accumulation of amino acids and ammonium is detected.^65^ Conversely, Timm and colleagues showed that when photorespiration is inhibited through HPR1 deletion, plants accumulate serine.^67^ The third and last known mechanism for serine biosynthesis in plants is the glycerate pathway.^66^ This metabolic route occurs in the cytoplasm in parallel with glycolysis and requires glycerate and alanine. We did monitor exacerbated levels of both glycerate and alanine in parallel with decreased carbohydrate in the *micu-1* context under these conditions. Hence, it is tempting to speculate that exacerbated calcium signaling activates serine biosynthesis through all serine biosynthesis pathways. Such alterations in serine levels are also likely to further contribute to the changes in metabolism described herein.

**Figure 5.**
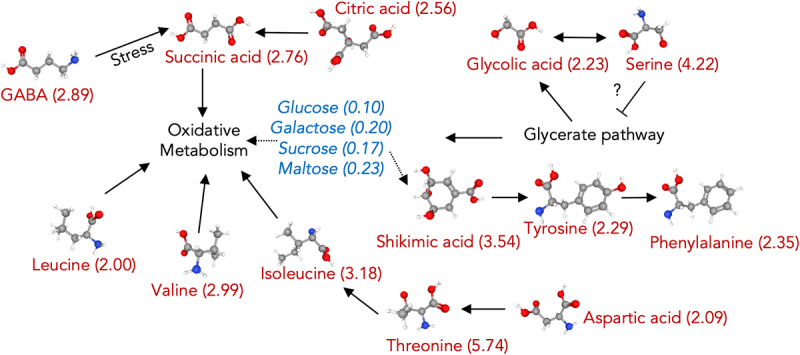
Potential metabolic consequences derived from genetic elimination of AtMICU. the levels of selected metabolites in *micu-1* plants as compared with Col-0 controls are shown. Upregulated metabolites are shown in red alongside its fold changes in parenthesis. Downregulated metabolites are shown in blue alongside its fold change decrease. Dotted lines represent reduced metabolic fluxes. Proposed metabolite fluxes in *micu-1* plants were retrieved from the KEGG metabolite library and from selected sources.^66,68^

The potential metabolic consequences derived from genetic elimination of AtMICU are summarized in Figure 5 and can be described as follows: In the presence of exacerbated calcium signaling due to the absence of AtMICU, oxidative metabolism is increased yielding reduced levels of carbohydrates, enhanced TCA cycle intermediates and other end products including amino acids. Indeed, Wagner and collaborators have detected an increased capability of *micu-1* plants to oxidize pyruvate/malate.^30^ Furthermore, the accumulation of signaling amino acids such as GABA could contribute to a constitutive state characterized by altered growth likely through regulating the expression levels of anion transporters including those for malate.^45,69^ Whether these transporters or other proteins involved in the transport or metabolism of citrate or succinate -both of which were found at enhanced levels in *micu-1* plants- remains to be tested. The results presented herein provide initial insight into the link between MCU-dependent calcium transport and its impact on the metabolism of *A. thaliana*. Further characterization of such changes at different ‘omic’ levels and on different genetic backgrounds is a work in progress in our laboratories.

## Supplementary Material

Supplemental Material

## Abbreviations

MCU: mitochondrial calcium uniporter
MCUc: mitochondrial calcium uniporter holocomplex
MICU: mitochondrial calcium uniporter regulator
PAB: protoplast assay buffer
PCA: principal component analysis
PLS-DA: partial least squares-discriminate analysis
PES: protoplast enzyme solution
ROS: reactive oxygen species
RuR: ruthenium red
TCA: tricarboxylic acid cycle
