# Supplementary figures and images for: An Arabidopsis mutant line lacking the mitochondrial calcium transport regulator MICU shows an altered metabolite profile
Source: Plant Signal Behav. 2023 Oct 25;18(1):2271799. doi: 10.1080/15592324.2023.2271799 (PMC10601504; doi:10.1080/15592324.2023.2271799)

A

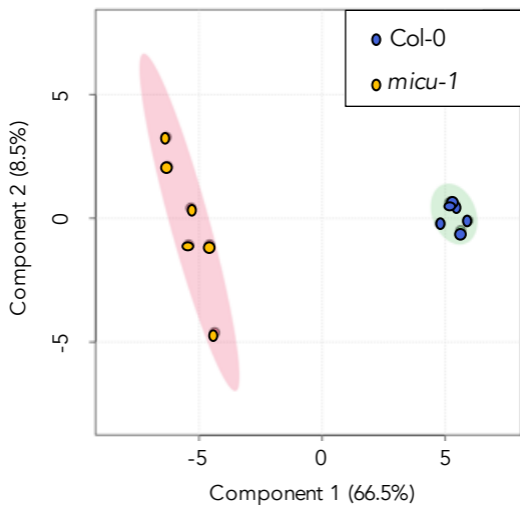

B

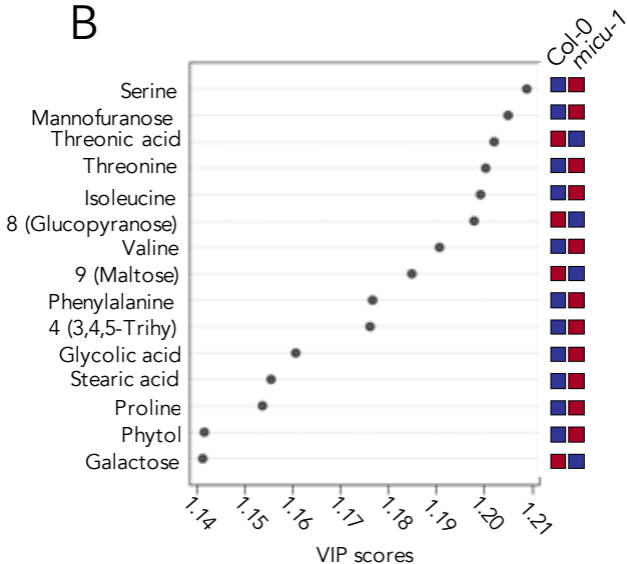

Supplement: Supplemental Material [file KPSB_A_2271799_SM1677.zip › Fig S2.pdf]

Scores Plot

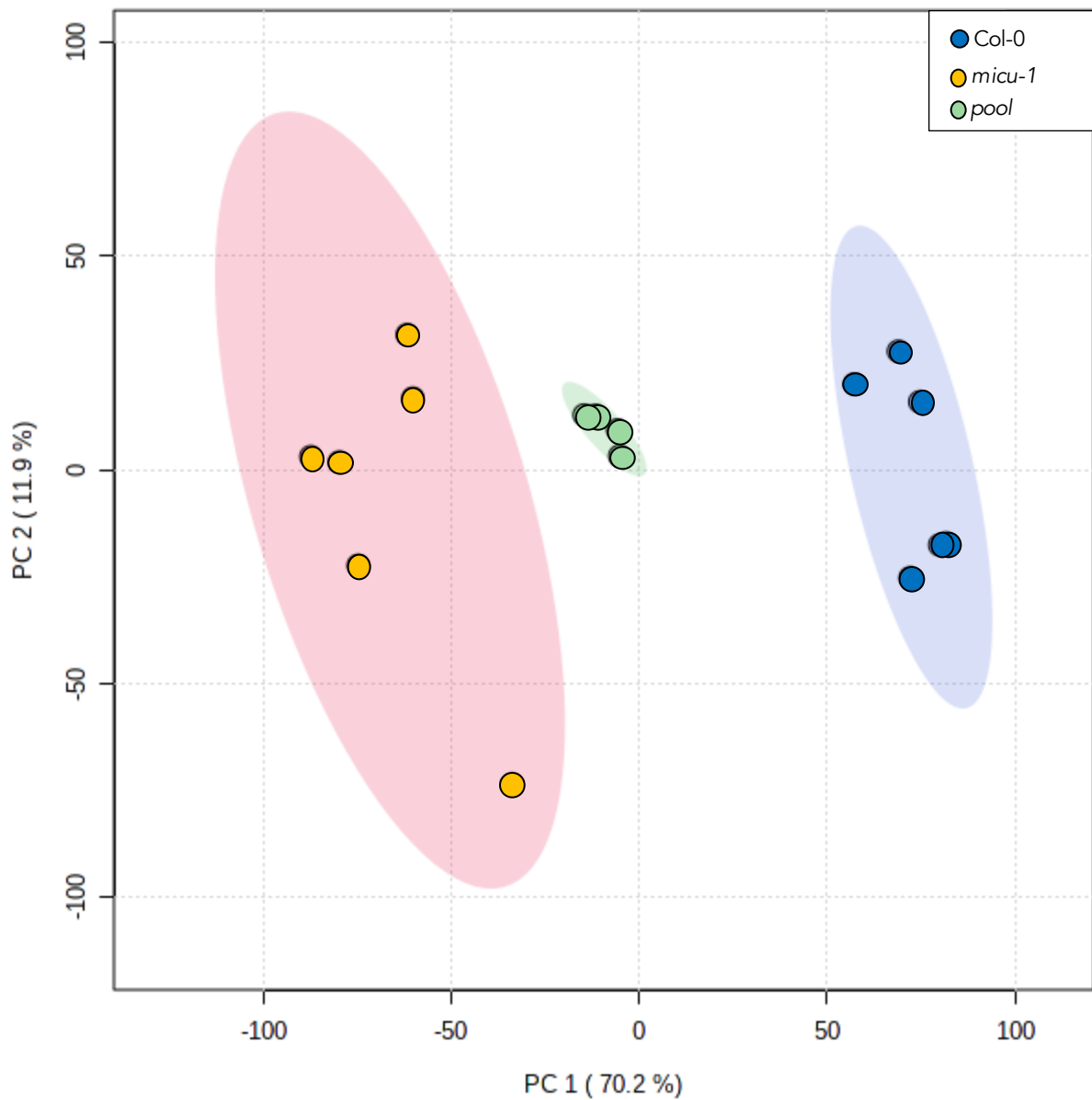

Supplement: Supplemental Material [file KPSB_A_2271799_SM1677.zip › Fig S3.pdf]
